# Investigation of the prevalence of Cognitive Impairment and its risk factors within the elderly population in Shanghai, China

**DOI:** 10.1038/s41598-018-21983-w

**Published:** 2018-02-23

**Authors:** Longbing Ren, Yongtao Zheng, Lezhou Wu, Yijun Gu, Yusheng He, Bo Jiang, Jie Zhang, Lijuan Zhang, Jue Li

**Affiliations:** 1 Shanghai East Hospital, Key Laboratory of Arrhythmias, Ministry of Education, Tongji University School of Medicine, Tongji University, Shanghai, 200120 China; 2 Institute of Clinical Epidemiology and Evidence-based medicine, Tongji University School of Medicine, Shanghai, 200092 P.R. China; 3 Department of Data Science, Children’s Hospital of Philadelphia, Philadelphia, PA 19147 USA; 4 Department of Neurology, Tongji Hospital, Tongji University, Shanghai, China

## Abstract

To investigate the prevalence of cognitive impairment and its risk factors among Chinese elders aged over 80 years, a community-based, cross-sectional study was conducted from May to June 2016 in Shanghai, China. Cognitive function was measured by using Mini-Mental Status Examination. Multiple logistic regression assessed associations between risk factors and cognitive impairment. Of 480 participants, 30% were diagnosed with cognitive impairment. Women [adjusted odds ratio (AOR): 1.71, 95% confidence interval (CI): 1.03–2.83], solitary life (AOR: 3.15, 1.89–5.26), monthly income less than 2000 Chinese yuan (AOR: 3.47, 1.18–10.23) were significantly associated with increased risk of cognitive impairment, compared with men, non-solitary life, and monthly income greater than 4000 Chinese yuan, respectively. Overweight (AOR: 0.59, 0.36–0.97), being physically active at least 60 minutes per day (AOR: 0.59, 0.35–0.95), antihypertensive drugs users (AOR: 0.45, 0.28–0.72), and lipid lowering drugs users (AOR: 0.21, 0.06–0.76) significantly lowered the risk of cognitive impairment, compared with normal weight, inadequate outdoor activity, and non-medication users, respectively. Accordingly, this study found that women, solitary life, lower income was associated with increased risk of cognitive impairment, while overweight, being physically active, and antihypertensive and lipid lowering drugs usage might lower the risk.

## Introduction

The elderly population such as those aged 80 years and over comprise the fastest growing segment of the global population^1^. They are more susceptible to most diseases than younger adults and therefore were the leading consumers of healthcare services^2^. Currently, how to prevent diseases related to aging is one of the greatest challenges in the healthcare field.

Cognitive impairment is one of the most common health problems for elders. It is estimated that the prevalence of cognitive impairment was higher than 40% among elders aged 80 years and over^3,4^. Cognitive impairment includes mild cognitive impairment and various types of dementia, and is associated with an increasing risk of disability and death^5^. Due to an unprecedented increase in life expectancy, the global prevalence of cognitive impairment is expected to grow exponentially in the coming years. Cognitive impairment not only causes a significant decline in the quality of life for patients, but also was a substantial economic burden for patients’ families and society in general. In Mainland China, the annual healthcare cost of cognitive impairment is estimated to be at least 9 billion U.S. dollars, while approximately 35 million individuals including patients and their families are either directly or indirectly affected by the deleterious effects of cognitive impairment^6^. Moreover, there is very few home-based assistance for patients with cognitive impairment in China; only 2% of affected families were able to directly take care of patients^6^. Accordingly, cognitive impairment is a crucial public health issue in China and effective preventive strategies are needed.

A key part of preventive strategies for cognitive impairment is to identify intervenable risk factors. It has been demonstrated that aging, family history of cognitive impairment, low levels of physical activity, less education, and the presence of epsilon 4 allele of the apolipoprotein E (APOE ε4) gene are major risk factors of cognitive impairment^7^. In addition, recent studies revealed a mechanism linking the occurrence of cognitive impairment to β-amyloid deposition, which might lead to gray matter atrophy and memory impairment^8,9^. While population health studies have investigated risk factors for cognitive impairment, however, this is still understudied in the Chinese population. Also, early research in cognitive impairment primarily studied elders aged between 65 and 80 years^10,11^, and a few even excluded those aged over 80 years^12^. But lately, as the highest-risk population for cognitive impairment and the fastest-growing population segment, the elderly population aged 80 years and over was increasingly suggested to be studied as an independent population^13^. Therefore, evidence from previous studies is still less conclusive regarding risk factors of cognitive impairment among Chinese elders aged 80 years and overs.

We conducted a cross-sectional study in a Chinese community-dwelling elderly population. This study aimed to (1) estimate the prevalence of cognitive impairment and potential risk factors among Chinese elders aged over 80 years, and (2) assess whether these risk factors independently predicted the risk of cognitive impairment.

## Methods

### Study population

A cross-sectional study was conducted from May to June 2016 in Shanghai, China. The target population of this study were those who were at least 80 years old, locally resided in Shanghai, and had normal hearing, vision and speech. Exclusion criteria included: (1) absence of cognitive function or in a vegetative state; (2) diagnoses of schizophrenia or serious mental retardation; (3) residence outside Shanghai. Potential participants were informed by telephone using primary care registration information or notified by community bulletin board. A total of 480 eligible elderly individuals were enrolled in this study, and underwent face-to-face interview and cognitive assessment. The study was approved by medical ethical review committee of Tongji University and informed consent was obtained for all participants. We confirm that all methods were performed in accordance with the relevant guidelines and regulations.

### Sociodemographic and Health-related Data

An interviewer-administered questionnaire was used to collect sociodemographic and health-related data including gender, age, education, marriage, personal income, smoking and drinking habits, and physical activities. Self-reported medical conditions were recorded including hypertension, diabetes, dyslipidemia, coronary heart disease, chronic obstructive pulmonary disease, tumor, and chronic kidney disease. Medication history was also self-reported including antidiabetic, antihypertensive, anticoagulation, diuretic, and lipid lowering drugs. Education attainment was summarized as elementary school or less (≤6 years education), middle school (6–9 years education), and at least some high school (>9 years of education). Marital status was measured with four response categories including married or cohabitating, never married, widowed, and divorced or separated; marital status was further dichotomized as solitary vs. non-solitary life. Personal income was categorized into ≤2000, 2000 to 4000, and >4000 Chinese Yuan per month. Physical activity was dichotomized as <60 minutes vs. ≥60 minutes outdoor activity per day (e.g., walk, shopping and so on). Weight and height was measured and Body Mass Index (BMI) was calculated as weight in kilograms divided by height in meters squared (kg/m^2^); BMI <24 and ≥24 kg/m^2^ were categorized as normal and overweight, respectively.

### Assessment of Cognitive Function

Cognitive function was assessed using Chinese versions of the Mini-Mental State Examination (MMSE) and the Montreal Cognitive Assessment (MoCA) in the pilot study. Both instruments have been shown valid and reliable among Chinese who are culturally and linguistically different from westerners^14,15^. The assessments were conducted strictly following the guidelines and protocols from the instrument advisory committees^16,17^.

The MMSE is a 30-point questionnaire measuring five cognitive domains including orientation (e.g., orientation to time and place), registration (e.g., repeating named prompts), language and praxis, attention and calculation, and recall. The raw total score need to be adjusted for education attainments when diagnosing severity of cognitive impairment^14^.

The MoCA is another 30-point questionnaire assessing eight areas of cognitive function including attention, concentration, executive functions, memory, language, visuoconstructional skills, conceptual thinking, calculations, and orientation. A total score of 26 points or higher indicates normal cognition^18^.

Our pilot study indicated a high proportion of low-educated participants (this was also later confirmed by the formal study: only 10% participants received some high school education while 64% participants only attended elementary school or even less). Accordingly, the MMSE was finally used as a measurement of cognitive function in the formal study, since the MMSE is able to assess cognitive function after factoring in individual educational level whereas the MoCA has the limitation of education bias if the raw score cannot be appropriately adjusted^19^. A total MMSE score less than 20 and 24 was therefore used to diagnose cognitive impairment, respectively for participants with only elementary school education and those having more than elementary school. The application of these two cut-off values has been well-documented among Chinese populations regarding acceptable sensitivity and specificity^14,15,20,21^.

### Quality Control

All investigators received standard training by neurologists and psychiatrists before data collection. Face-to-face interview and cognitive assessment (the MMSE and MoCA) were both performed on the same day.

### Reliability of the MMSE

The test-retest reliability of the MMSE was assessed in a random subsample (n = 30, 18 men and 12 women, age range 80–92 years, average age 85.1 years). The retest was completed in 6 months after the initial one. The test and retest MMSE scores were highly correlated (r = 0.840, p < 0.001). A paired t-test indicated no significant difference in MMSE scores between two tests (mean ± standard deviation, 23.33 ± 4.44 vs. 22.50 ± 5.14, p > 0.05). Other two reliability statistics were also calculated: coincidence rate equaled 90% and Kappa coefficient was 0.667 (p < 0.001). All evaluation above indicated substantial agreement between repeated MMSE.

**Figure 1:**
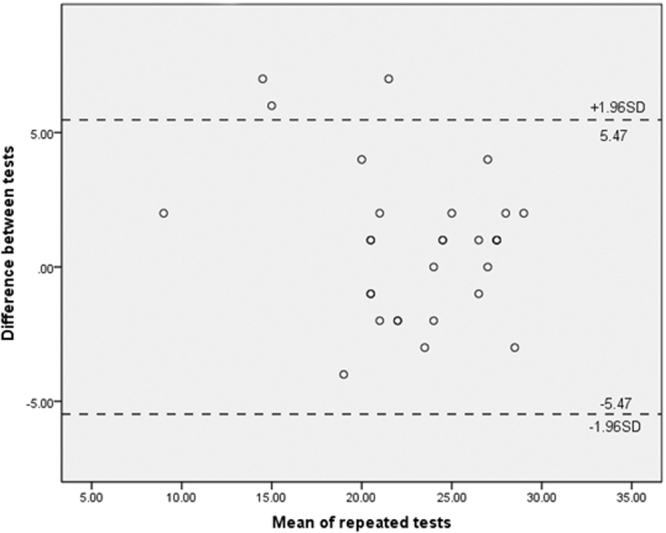
The Bland-Altman plot for the MMSE reliability assessment. The graph displays a scatter diagram of the test-retest differences plotted against the averages of the two tests. Horizontal dotted lines were drawn at the limits of agreement, which were defined as the mean difference ±1.96 times the standard deviation of the differences. For repeated measurements, the expected mean difference was set to zero.

In addition, a Bland-Altman plot was used to illustrate the MMSE reliability. The differences of two repeated measurements were plotted against means of repeated measurements. Two horizontal dotted lines then were added indicating 95% limits of agreement, which were correspondingly estimated by mean difference ±1.96 times the standard deviation of the differences (mean difference was expected to be zero). As shown in Fig. 1, approximately 90% of differences between repeated MMSE fell within the interval provided by 95% limits of agreement. Therefore, reliability of the MMSE was demonstrated.

### Statistical analyses

EpiData 3.1 (Odense, Denmark) was used for data input, and statistical software SPSS 20.0 (SPSS Inc., Chicago, IL, USA) was used for data management and analyses. The chi-squared test was used to analyze the crude differences between participants with cognitive impairment versus those with normal cognition. Multiple logistic regression analysis was applied to assess independent risk factors associated with increased risk of cognitive impairment; multivariable-adjusted odds ratio (AOR) and 95% confidence interval (CI) was calculated for each risk factor indicating the magnitude of cognitive impairment risk. Multiple correspondence analysis (MCA) was further conducted to present the association of potential risk factors with cognitive impairment. To further assess the domain-specific cognitive function, bar plots were produced presenting the differences in average domain-specific MMSE scores by gender and by cognitive impairment diagnosis. (For direct comparison, five domain-specific MMSE scores were transformed to a 0–100% scale: sum of the points earned divided by the total points possible.) Two-sided p value less than 0.05 was considered statistically significant.

## Results

Demographic and health characteristics of study participants by cognitive function status were summarized in Table 1. Of 480 participants, 29.9% were diagnosed with cognitive impairment. The majority of participants were female (55.3%), aged from 80 to 85 years (63.6%), solitary (51.6%), attended only elementary school or even less (64%), had a monthly income of between 2000 and 4000 Chinese yuan (54.8%), never smoked (80.4%), never drank (88.9%), had outdoor activity more than 60 minutes per day (50.8%), and had a normal BMI less than 24 (55.4%). The three most prevalent self-reported diseases were hypertension (57.2%), coronary heart disease (24.5%), and diabetes (16.4%). The three most commonly taken medications were antihypertensive (55.2%), anticoagulation (21.3%), and antidiabetic (14.4%) drugs. When comparing participants with cognitive impairment to those with normal cognitive function, there were significant crude differences in gender, marital status, personal income, physical activity, hypertension, antihypertensive usage, and lipid lowering drugs usage (all p < 0.05).

**Table 1:** Demographic and health characteristics of participants with cognitive impairment vs. normal cognition.

Risk factors	Cognitive Impairment (n = 144)	Normal Cognition (n = 336)	*p*-value
***Sociodemographic and behavioral features***			
**Gender**			<0.001
Men	41(28.5%)	174(51.8%)	
Women	103(71.5%)	162(48.2%)	
**Age (year)**			0.094
≤85	82(56.9%)	223(66.4%)	
≥86	59(43.1%)	112(33.6%)	
**Education**			
Elementary school or less	106(73.6%)	200(59.5%)	0.001
Middle school	33(22.9%)	91(27.1%)	
At least some high school	5(3.5%)	45(13.5%)	
**Marital Status**			<0.001
Solitary	107(74.3%)	141(42.0%)	
Non-solitary	37(25.7%)	195(58.0%)	
**Monthly income** (Chinese yuan)			0.001
≤2000	53(37.9%)	85(27.6%)	
2000–4000	82(58.6%)	181(58.8%)	
>4000	5(3.5%)	42(13.6%)	
**Body Mass Index** (kg/m^2^)			0.002
Normal (BMI <24)	93(64.6%)	173(51.5%)	
Overweight (BMI ≥24)	41(35.4%)	151(48.5%)	
**Smoking**			0.334
Never smoker	122(84.7%)	264(78.6%)	
Ever smoker	14(9.7%)	49(14.6%)	
Current smoker	8(5.6%)	23(6.8%)	
**Drinking**			0.161
Never drinker	134(93.1%)	293(87.2%)	
Ever drinker	7(4.9%)	27(8.0%)	
Current drinker	3(2.0%)	16(4.8%)	
**Physical activity**			0.006
<60 min per day	85(59.0%)	151(44.9%)	
≥60 min per day	59(41.0%)	185(55.1%)	
**Medical history**			
Hypertension	70(48.6%)	205(61.0%)	0.016
Diabetes	26(18.1%)	53(15.8%)	0.502
Dyslipidemia	13(9.0%)	49(14.6%)	0.135
Coronary heart disease	28(19.4%)	90(26.8%)	0.105
Chronic obstructive pulmonary disease	8(5.6%)	11(3.6%)	0.306
Tumor	2(1.4%)	12(3.6%)	0.314
Chronic kidney disease	2(1.4%)	11(3.3%)	0.314
Other diseases	13(7.1%)	16(4.8%)	0.246
**Medications**			
Antidiabetic	23(16.0%)	46(13.7%)	0.514
Antihypertensive	60(41.7%)	205(61.0%)	<0.001
Lipid lowering drugs	3(2.1%)	32(9.5%)	0.004
Anticoagulation	24(16.7%)	78(23.5%)	0.105
Diuretics	5(4.2%)	17(5.1%)	0.491

Categorical variables were presented as count and column percent.

The adjusted associations between risk factors and the risk of cognitive impairment were shown in Table 2. Gender, marital status, personal income, BMI, physical activity, antihypertensive usage, and lipid lowering drugs usage were found to be independent predictors for cognitive impairment risk (all p < 0.05). After controlling for confounding, women were more likely than men to have cognitive impairment (AOR: 1.71, 95% CI: 1.03–2.83); solitary life (AOR: 3.15, 95% CI: 1.89–5.26) and monthly income less than 2000 Chinese yuan (AOR: 3.47, 95% CI: 1.18–10.23) was significantly associated with increased risk of cognitive impairment, compared with non-solitary life and monthly income greater than 4000 Chinese yuan, respectively. In contrast, being overweight (AOR: 0.59, 95% CI: 0.36–0.97) and physically active at least 60 minutes per day (AOR: 0.59, 95% CI: 0.35–0.95) was both significantly associated with a more than 40% decrease in cognitive impairment risk, compared with normal weight and inadequate outdoor activity (less than 60 minutes per day), respectively. Also, antihypertensive (AOR: 0.45, 95% CI: 0.28–0.72) and lipid lowering drugs (AOR: 0.21, 95% CI: 0.06–0.76) usage lowered cognitive impairment risk. Age was not associated with cognitive impairment risk in this elderly Chinese population (AOR: 0.81, 95% CI: 0.49–1.34).

**Table 2:** Adjusted odds ratio for the risk of cognitive impairment.

Predictors	AOR	95% CI	*p*-value
**Gender** (women)	1.707	1.028–2.833	0.039
**Age** (≥86 years)	0.813	0.492–1.341	0.417
**Marital status** (solitary life)	3.152	1.889–5.261	0.000
**Monthly income**			
≤2000 Chinese yuan	3.472	1.178–10.231	0.024
2000–4000 Chinese yuan	1.721	1.031–2.875	0.038
**BMI** (overweight, BMI ≥24 kg/m^2^)	0.590	0.359–0.969	0.037
**Physical activity** (≥60 min per day)	0.580	0.354–0.951	0.031
**Antihypertensive drugs use**	0.447	0.277–0.721	0.001
**Lipid lowering drugs use**	0.212	0.059–0.764	0.018

Multiple logistic regression analysis estimated the risk of cognitive impairment associated with potentially independent risk factors. The reference groups for comparison were men, age ≤85 years, non-solitary life, monthly income >4000 Chinese yuan, BMI <24, physical activity <60 minutes per day, no use of antihypertensive drugs, and no use of lipid lowering drugs, respectively.

**Figure 2:**
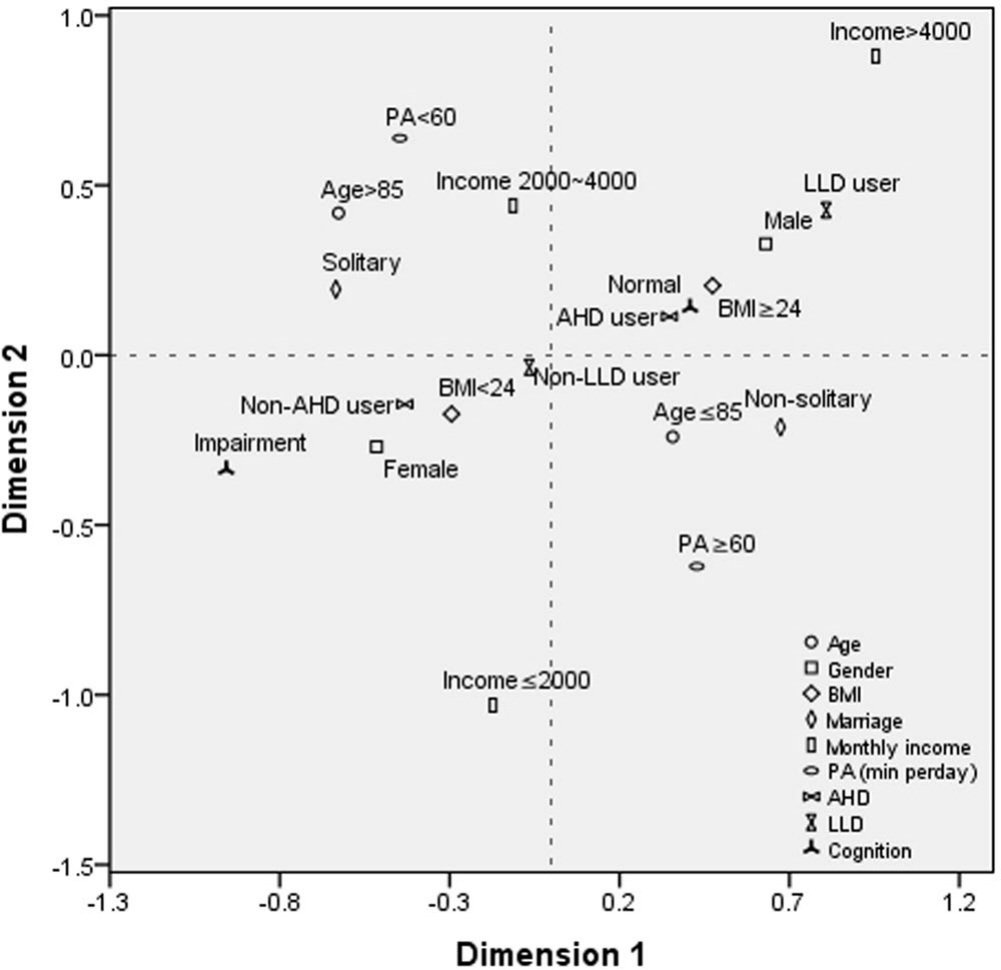
Multiple correspondence analysis (MCA) plot. Risk factors were presented as icons and a brief description was labeled near the icon indicating a specific category of risk factors. To interpret MCA plot, icons closed together in the same quadrants along the similar direction from centroid would be indicative of possible associations. BMI, body mass index; PA, physical activity; AHD, antihypertensive drugs; LLD, lipid lowering drugs.

The associations between risk factors and cognitive impairment were further presented using the MCA plot (Fig. 2). In the MCA plot, the associations are interpreted based on the distance between two variables in the same quadrant: when two variables appear in the similar direction from centroid, the closer distance suggested a more likely association^22^. As shown in Fig. 2, gender, BMI, antihypertensive and lipid lowering drugs were more likely to be associated with cognitive function in this Chinese elderly population. In contrast, age, marriage, monthly income, and daily physical activity were less likely to predict cognitive function. The MCA plot is a statistically descriptive technique and was applied as a supplement for regression analysis in this study.

**Figure 3:**
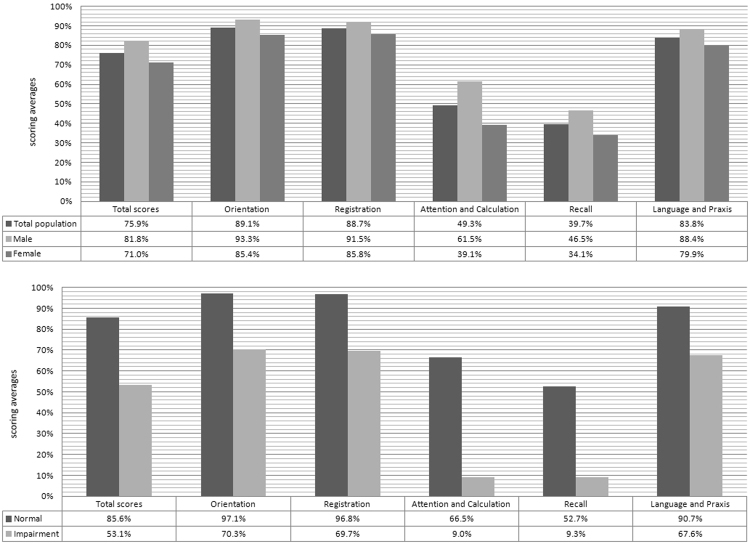
Domain-specific cognitive function by gender and diagnosis of cognitive impairment. Five domain-specific MMSE scores were transformed to a 0–100% scale (sum of the points earned divided by the total points possible). The scoring averages were plotted.

Transformed domain-specific MMSE scores stratified by gender and the diagnosis of cognitive impairment were presented in Fig. 3. In this elderly Chinese population, MMSE scores appeared to be obviously lowest in the cognitive domains of attention and calculation (49.3%), as well as recall (39.7%). This disparity remained when assessing women and men separately. Moreover, domain-specific MMSE scores were consistently lower among elders with cognitive impairment than among those with normal cognition; however, the differences in MMES scores were remarkably large in the attention and calculation domain (66.5% vs. 9.0%), as well as recall domain (52.7% vs. 9.3%).

## Discussion

Cognitive impairment is an aging-related disease, which can result in various health problems, including disability and death. It has been reported that the prevalence of cognitive impairment is greater than 40% among elderly individuals^3^. Cognitive impairment is not only highly prevalent, but has a great impact on life quality, thus imposing a substantial socioeconomic burden. Cognitive impairment is now a major public health concern in Mainland China^23^, and to identify potential risk factors of cognitive impairment is fundamental for developing preventive strategies.

We conducted a community-based, cross-sectional study to assess independent predictors for the risk of cognitive impairment. Our findings showed that approximately 30% of participants aged 80 years and over were diagnosed with cognitive impairment, with a prevalence of 19% among men and 39% among women. Women were further found to be associated with increased risk of cognitive impairment after controlling for confounding. This is consistent with a previous study wherein male patients with Alzheimer’s disease outperformed female counterparts in cognitive function^24^. A possible explanation for this female disadvantage was hormonal influence: the decrease in estrogen level associated with aging may adversely affect cognitive function among women^24^.

Another independent risk factor for cognitive impairment was marital status: solitary elders had a threefold higher risk of cognitive impairment than those having a non-solitary life. Solitary individuals often have less opportunity to communicate and are less involved in social activities, and thus lower social engagements may result in higher risk of cognitive impairment^25^.

Lower personal income was also found predictive of increased risk of cognitive impairment. There are several explanations for this relationship: (1) lower income is associated worse nutritional intake, such as less consumption of dairy products^26^; (2) lower socioeconomic status is associated less access to healthcare services^2,27^; (3) lower socioeconomic status is also associated with less social activity and less interpersonal communication, and lack of income can even lead to social isolation^25,28^.

Other factors appeared to reduce the risk of cognitive impairment including overweight, being physically active, and antihypertensive and lipid lowering drugs usage. Overweight (BMI >24 kg/m^2^) was associated with a 40% reduction in cognitive impairment risk compared with normal weight, which was consistent with a previous study in a Finnish population^29^. Higher BMI reflects increased body fat^30,31^. Body fat such as leg fat has been demonstrated to improve glucose metabolism^32^, and better cerebral glucose metabolism might lower the risk of cognitive impairment^33^. In addition, since cognitive function is closely correlated to nutrition^34^, low body fat among elders might impair cognitive function via malnutrition^35,36^.

Increased daily physical activity was found associated with reduced risk of cognitive impairment independent of other confounders. The benefits of being physically active for cognitive impairment among elders has been widely studied^37^. Randomized controlled trials even assessed exercise-based interventions, indicating physical activities effectively lower the risk of dementia and improve multiple aspects of cognitive function^38^. Our analysis further confirmed this finding among individuals aged over 80 years, and suggested the oldest population can still significantly benefit from daily physical activity for their cognitive function.

We also found antihypertensive and lipid lowering drugs usage were independent protective factors for cognitive function. Population health studies inconsistently reported the relationships between serum cholesterol level and cognitive function among elders^39,40^. A recent study indicated a decreased risk of cognitive impairment associated with higher serum cholesterol levels^39^; in contrast, earlier research found significantly higher cholesterol levels among elderly individuals with cognitive impairment, and suggested cholesterol lowering therapy such as lipid lowering drugs might prevent cognitive impairment beyond its cardiovascular benefit^41,42^. Our findings now confirmed that lipid lowering drugs usage might lower the risk of cognitive impairment among Chinese elders aged over 80 years. Evidences for possible biological mechanisms also linked high cholesterol level to neurodegenerative diseases^43^: abnormal cholesterol metabolism increased the production and deposition of β-amyloid in the brain, and in turn lead to cognitive impairment^8,9^; cholesterol-carrying proteins also markedly increase the risk of cognitive impairment^43^. Despite such evidence, randomized clinical trials are still needed to clarify these inconsistent findings.

The present study demonstrated the protective effect by antihypertensive drugs for cognitive impairment. This confirmed previous findings: antihypertensive treatment was found a 50% reduction in dementia incidence in an elderly population^44^; among hypertensive patients, those having antihypertensive treatment had significantly better cognitive performance than untreated counterparts^45^; poorer blood pressure control was associated with higher risk of cognitive impairment among patients who have been treated for hypertension^46^. These benefits can be explained by the fact that antihypertensive drugs prevent or slow brain white matter lesions caused by high blood pressure.

Additionally, previous studies have reported that smoking, alcohol abuse, diabetes, and hyperlipidemia might be risk factors of cognitive impairment in all age groups, and especially for those aged 65 years and over^21,47–49^. However, none of these were identified in this elderly Chinese population aged over 80 years. This may be due to so called competing causes of death. Of 480 study participants, 81% have never smoked and 90% have never drunk, while only 16% were diabetic and 13% had dyslipidemia. It suggests elders with these risk factors might pass away before reaching the age 80 years.

There are limitations to this study that should be noted. First, a cross-sectional study may not fully assess the temporality between risk factors and cognitive outcome, and thus the causal relationship is still indeterminate. A longitudinal follow-up study is needed to address the limitation. Second, the sample size of this study may not provide adequate statistical power to detect significant difference, especially for categorical variables with rare events. Third, this study utilized the MMSE rather than gold standard measures^50^ (e.g., DSM-111, NINCDS-ADRDA, and clinical record) to evaluate cognitive function, and thus we were unable to diagnose dementia. Also, there might be more measurement bias comparing the MMSE with gold standard measures, but the reliability and validity of the MMSE have been well assessed among different populations including elderly Chinese^14,15,20,21,51^. Finally, blood samples were not collected in this study and therefore laboratory indicators such as blood glucose and lipid could not be measured. Self-reported medical conditions were less valid than that diagnosed using laboratory measures. This may also result in possible residual confounding when assessing adjusted association between risk factors and cognitive impairment.

## Conclusion

Approximately 30% of Chinese elders aged 80 years and over were diagnosed with cognitive impairment. The risk of cognitive impairment was independently associated with risk factors such as gender, marital status, personal income, BMI, physical activity, and antihypertensive and lipid lowering drugs usage: women, solitary life, lower income was associated with increased risk of cognitive impairment, while overweight, being physically active, and antihypertensive and lipid lowering drugs usage was associated with lower risk of cognitive impairment. Among five specific cognitive functions, the domain of attention and calculation and recall domain were markedly weak for elders with cognitive impairment.
